# The elimination of human African trypanosomiasis is in sight: Report from the third WHO stakeholders meeting on elimination of gambiense human African trypanosomiasis

**DOI:** 10.1371/journal.pntd.0006925

**Published:** 2018-12-06

**Authors:** Michael P. Barrett

**Affiliations:** Wellcome Centre for Molecular Parasitology, Institute of Infection, Immunity and Inflammation, University of Glasgow, Glasgow, United Kingdom; International Centre of Insect Physiology and Ecology, KENYA

In 2012, the World Health Organisation (WHO) set out a roadmap for the control, elimination, or eradication of 17 neglected tropical diseases by 2020 [1]. Many were skeptical about the achievability of such goals. Now, still two years away from that end point, good news is emerging for gambiense human African trypanosomiasis (HAT), or sleeping sickness, caused by the tsetse-fly−transmitted protozoan parasite *Trypanosoma brucei gambiense* in West and Central Africa [2]. The Rhodesiense form of the disease is being pursued under a separate programme.

This was the conclusion of the third WHO meeting of stakeholders on the elimination of gambiense HAT in Geneva between the 18th and 20th of April 2018, attended by representatives from national sleeping sickness control programmes, groups developing new tools to fight HAT, international and nongovernmental organisations involved in HAT control, industrial partners (Bayer Healthcare, Sanofi, and Vestergaard), and funding agencies (including the Belgian Development Cooperation and the Bill and Melinda Gates Foundation). Things have moved on substantially since the inaugural meeting of this group in 2014 [3].

The notional global target of <2,000 reported HAT cases by 2020 has already been met. Preliminary figures collated by WHO indicate that fewer than 1,500 cases were reported in 2017, although some foci remain above the threshold of 1 case or fewer per 10,000 people, and there are still some endemic areas where accessibility is limited. Reported incidence has diminished by >95% since the turn of the century [4]. This extraordinary success story is bringing the gambiense HAT elimination programme into the realms of some of the more celebrated—and better funded—programmes, such as those against Guinea worm [5] and polio [6], diseases that have been vying for the position of second human affliction, after smallpox, to be eradicated.

In spite of the success, HAT does, however, remain a public health problem in some countries in which various factors conspire to limit progress. Take Guinea, for example, where the Ebola outbreak of 2014 through 2015 led to a cessation of HAT control activities and an increase in the HAT burden [7]. Appropriate funding, ownership of the HAT problem by endemic countries, and political and social stability will all be key to ensure further progress and avoid setbacks.

History tells us that successful elimination campaigns can become self-limiting when national health authorities struggle to justify sustained investment in the face of dwindling case numbers while confronting other, higher-burden diseases. Efforts are, therefore, required to galvanise endemic countries and the international community to maintain commitment to the elimination goals. It will be necessary, for example, to ensure continued staff training, awareness, and motivation in the face of the falling burden of HAT. Integrating control, surveillance, and management of HAT into strengthened national health systems, in place of the autonomous specialised HAT programmes, offers a possible means to achieve this. New tools emerging in the fight against HAT may help underpin this transition.

For example, orally available drugs that can treat stage 2 disease (when parasites are found within the central nervous system) are appearing for the first time. The Drugs for Neglected Diseases initiative (DNDi) has recently completed successful clinical trials with fexinidazole [8], which is effective after a 10-day course of once-daily tablets. Sanofi will produce the drug which was recently approved by the European Medicine’s Agency, and is committed to ensure access to this drug via a donation to WHO for free distribution. A second oral treatment, acoziborole (AN5568 or SCYX-7158) [9], which has the advantage of being administered as a single 960 mg dose of three tablets, is currently in phase 2/3 clinical trials with DNDi. Other promising new compounds are currently completing preclinical development by the Novartis Institute of Tropical Diseases.

Control of the tsetse fly vector of HAT has also been central to integrated approaches [10], and the implementation of insecticide impregnated nets, the so-called tiny targets [11], has been of great utility, especially when coordinated with active case-finding campaigns and drug treatment of patients. Vector targeting also interfaces with control of animal trypanosomosis, and possible synergy at the One Health interface could offer the means to precipitate the downfall of both human and animal African trypanosomiases [12].

A major concern, as the incidence plummets, is how to focus case detection efforts in areas where the disease is still active. National programmes and external agencies balk at the idea of screening millions of people in regions where the disease is now absent or presents a very low prevalence. The impetus must therefore fall to the national programmes, with WHO support, to enhance integration in general healthcare systems, enhance community input in case-finding, and guide active case-detection efforts where the disease persists.

The reduction in case numbers is bringing other challenges too. For example, the positive predictive value of any diagnostic test diminishes as the disease burden drops, and this is being seen with the serological tests available for HAT, both the classical card agglutination test for trypanosomiasis (CATT) test and also the recently introduced rapid tests [13]. New, more specific tests are needed, perhaps expanding the range of antigens used in immunoassays or using protein, nucleic acid, or metabolite biomarkers [14].

Programmes including the European Union (EU)-funded DiTECT HAT initiative which aims at systematic evaluation of diagnostic tests for HAT, through the Institut de Recherche pour le Developpement (IRD), the Foundation of Innovative New Diagnostics (FIND)’s programme of testing, and other initiatives through the Institute of Tropical Medicine (ITM) are helping the evaluation of new tools and screening protocols.

Currently, the passage to market use of any diagnostic test ultimately requires commercial partners. However, economic incentives that encourage investment in new tests for a disease heading towards elimination are non-existent, thus leading to a real lack of access of diagnostic tools in HAT-endemic areas. A potential solution to this diagnostics problem would be to emulate the success that has assured access to medicines for neglected diseases [15], with similar international commitment for new diagnostic tools.

Other challenges are becoming apparent only as we move towards elimination of the disease. For example, roles of asymptomatic human carriers and animal reservoirs [16] in HAT epidemiology require further attention. Both adipose tissue [17] and skin [18,19] have recently been shown to be sites of proliferation of trypanosomes and might explain the occurrence of serologically positive patients who do not yield trypanosomes in the blood.

Notwithstanding these issues, with the current tools, success has been such that WHO has established a formal protocol that enables any country endemic for HAT to claim for elimination of the disease as a public health problem and receive WHO validation.

The meeting contemplated how the phenomenal successes achieved in the campaign to defeat HAT could be brought to the attention of the world. A high-profile ambassador to disseminate information regarding the campaign may bring great benefits. There is no doubting the importance of former United States president Jimmy Carter, for example, as a figurehead for the Guinea worm eradication campaign. Someone with similar personal charisma, passion, and global renown would be ideal to spearhead the efforts to achieve and sustain the elimination of HAT.
